# Discussion on machine learning technology to predict tacrolimus blood concentration in patients with nephrotic syndrome and membranous nephropathy in real-world settings

**DOI:** 10.1186/s12911-022-02089-w

**Published:** 2022-12-20

**Authors:** Weijia Yuan, Lin Sui, Haili Xin, Minchao Liu, Huayu Shi

**Affiliations:** 1 Department of Information, Medical Supplies Center of PLA General Hospital, Beijing, China; 2 Department of Pharmacy, Medical Supplies Center of PLA General Hospital, Beijing, China

**Keywords:** Blood concentration prediction, Machine learning, Tacrolimus, Nephrotic syndrome, Membranous nephropathy

## Abstract

**Background:**

Given its narrow treatment window, high toxicity, adverse effects, and individual differences in its use, we collected and sorted data on tacrolimus use by real patients with kidney diseases. We then used machine learning technology to predict tacrolimus blood concentration in order to provide a basis for tacrolimus dose adjustment and ensure patient safety.

**Methods:**

This study involved 913 hospitalized patients with nephrotic syndrome and membranous nephropathy treated with tacrolimus. We evaluated data related to patient demographics, laboratory tests, and combined medication. After data cleaning and feature engineering, six machine learning models were constructed, and the predictive performance of each model was evaluated via external verification.

**Results:**

The XGBoost model outperformed other investigated models, with a prediction accuracy of 73.33%, F-beta of 91.24%, and AUC of 0.5531.

**Conclusions:**

Through this exploratory study, we could determine the ability of machine learning to predict TAC blood concentration. Although the results prove the predictive potential of machine learning to some extent, in-depth research is still needed to resolve the XGBoost model’s bias towards positive class and thereby facilitate its use in real-world settings.

## Background

Tacrolimus (TAC, FK506) is a new immunosuppressant that functions by inhibiting the activity of calcineurin and interfering with T cell activation and cytokine transcription after binding to intracellular FK binding protein. Recent studies have shown that TAC is effective in the treatment of a variety of chronic kidney diseases [1, 2]. However, its narrow treatment window, high toxicity, adverse effects, and individual differences in pharmacokinetics and pharmacodynamics have hindered its application in clinical treatment. Therefore, in clinical use, monitoring the blood concentration, adjusting the treatment plan, and administering individualized dosages of TAC are necessary to achieve the best treatment effect [3].

Real-world medical data are widely stored in hospital information systems, which include comprehensive diagnostic and treatment information. The optimization, upgrading, and popularization of hospital information systems not only provide a basis for the medical treatment of patients but also supply real-world data for retrospective research. Machine learning (ML) is a set of computer algorithms driven by data [4]. Its algorithms include the following: artificial neural network, decision tree, random forest, and support vector machine. ML is suitable for analyzing and mining real-world data in enormous quantities, high dimensions, complex relationships, and diverse forms. The rapid speed and strong generalizability of ML support its wide use in clinical decision-making. The application of ML algorithms to individualized medicine will aid in the understanding of precision medicine in clinical practice [5, 6]. The purpose of this study was to explore the influencing factors of TAC blood concentration in real-world settings using ML technology to predict TAC blood concentration and assist clinicians in adjusting TAC dosage, ensuring patient safety, and reducing adverse drug reactions.

## Methods

### Study population

The data of patients with nephrotic syndrome and/or membranous nephropathy treated with TAC in PLA General Hospital from January 1, 2013, to December 31, 2020, were collected retrospectively. The inclusion criteria were as follows: (1) diagnosis of nephrotic syndrome or membranous nephropathy; and (2) administration of TAC during hospitalization. The exclusion criteria were as follows: (1) use of TAC only during surgery; (2) TAC administration by skin test; and (3) patients with any missing data. This study was approved by the Ethics Committee of the Chinese People's Liberation Army General Hospital [S2022-278–01].

**Fig. 1:**
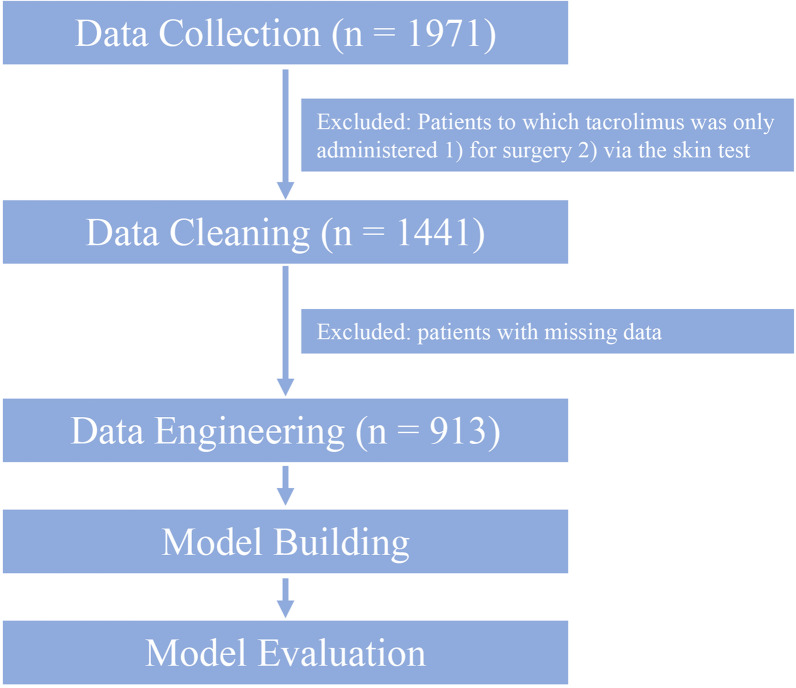
Flow chart of data mining and modeling

**Fig. 2:**
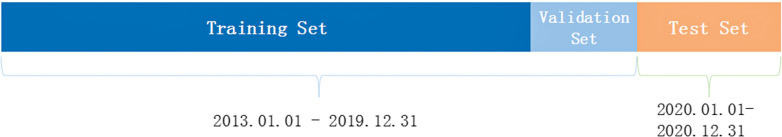
Division of datasets

The data mining and modeling processes are shown in Fig. 1. Following the cleaning step, the final data set comprised 913 patients and the blood TAC concentrations from 1829 blood tests. Data from January 1, 2013, to December 31, 2019, including 821 patients and 1,649 blood tests, were randomly divided into a training set and a test set at an 8:2 ratio. The data from January 1, 2020, to December 31, 2020, including 115 patients and 180 blood tests, were used as the external validation set (Fig. 2).

### Data extraction

**Fig. 3:**
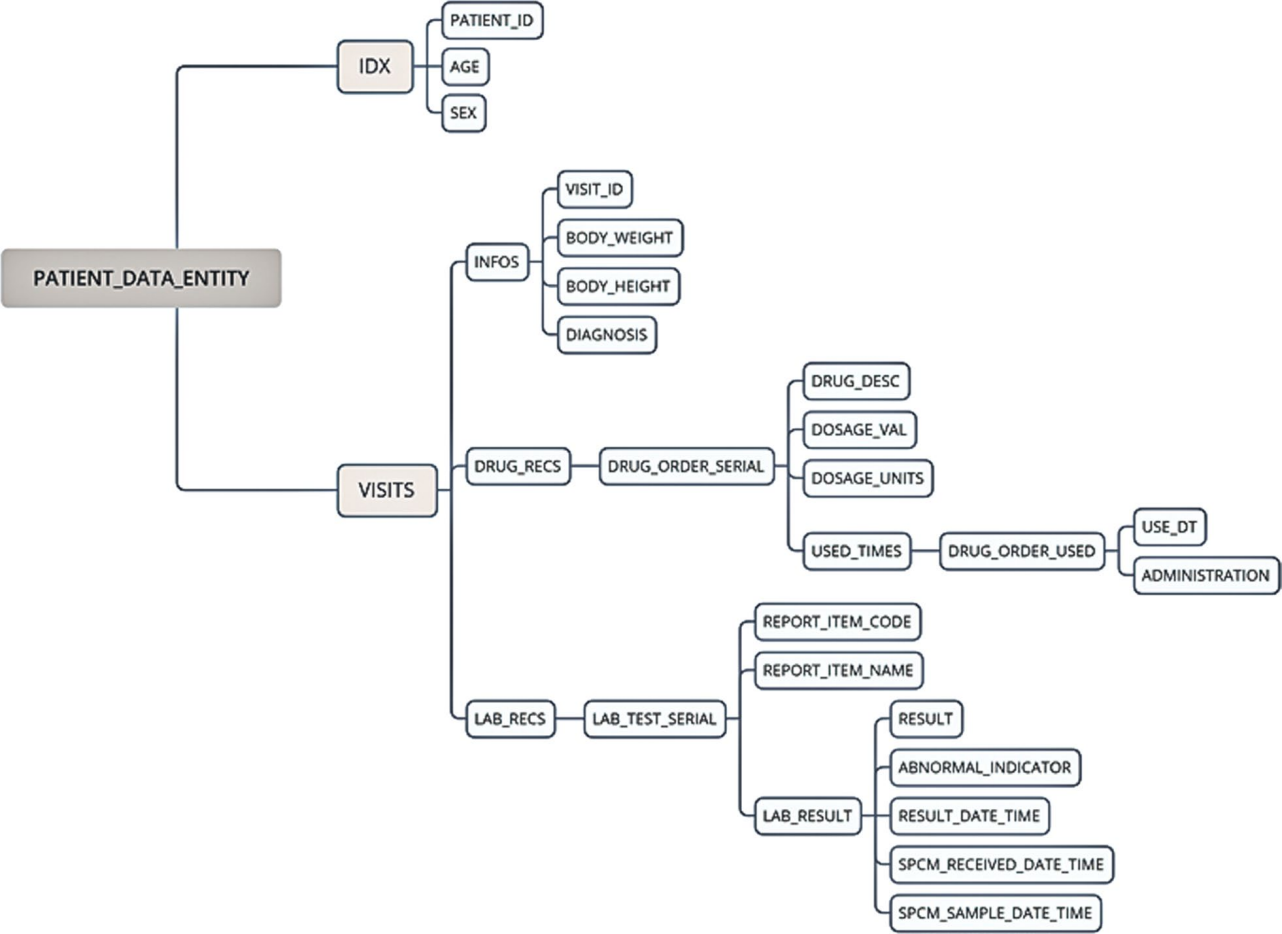
Patient information in a tree structure

The relevant patient information was extracted from the database, including demographic, laboratory, and medical order information. Demographic information included data on age, sex, height, and weight. The laboratory information included the blood TAC concentrations, serum creatinine levels, sample receiving times, and result indicators. The medical order information included the name of the medication, dose, frequency of administration, and start and end times of the treatment. Because the medical order consisted of long-term information, it was split by frequency and processed into time-series data. To facilitate data processing, we stored patient hospitalization information in a tree structure rather than a two-dimensional table to build the data set (Fig. 3).

### Data processing

First, data distribution was drawn according to the demographic information, and samples with outliers were deleted. Second, the medication and laboratory information were associated according to time. When there were multiple administrations of TAC before the collection of blood samples, we selected the data from the last TAC administration before sample collection to ascertain the test results matched the corresponding TAC administration. Additionally, a box plot was drawn for the time interval between the last administration and sample-receiving time, and only the samples between quartile 1 (Q1) and quartile 3 (Q3) were reserved to eliminate samples whose medication information was not related to the laboratory information. Seven doses of TAC were administered, and we organized them by the frequency of use as follows: 2.0, 1.0, 1.5, 3.0, 0.5, 2.5, and 4.0 mg.

In terms of combined medication, we extracted information on some of the most commonly prescribed medications by clinicians, including compound α-ketoacid, Shenyankangfu, ShenYanShu, Shenshuaining, Huangkui, Bailing, pidotimod, methylprednisolone, prednisone acetate, mycophenolate mofetil, and tripterygium glycoside. The variables of combined medication were dummy variables. Patients who had used one of these drugs between two blood tests were recorded as 1, and those who did not were recorded as 0.

Although blood concentration is a continuous variable, it was treated as a dummy variable in this study and classified according to the safe range of blood drug concentration [7, 8]. Concentrations were defined as 0 within the safety range, and those outside the safety range were defined as 1.

In this study, the blood concentration ratio of TAC classes 0 to 1 was unbalanced at 3:7. Therefore, we used the over-sampling method, SMOTE (Synthetic Minority Oversampling Technique), to balance the data. The core of SMOTE is to insert randomly generated new samples between those of minority and adjacent categories to increase the number of minority categories and improve the unbalanced distribution of the data set [9]. As XGBoost and LightGBM (LGBM) algorithms have hyperparameters for processing unbalanced data, we directly adjusted the super parameters without additional SMOTE processing of data for these algorithms.

### Feature selection

The extracted variables included demographic information (age, sex, height, and weight), laboratory information (numerical results and collection time of blood TAC concentration and serum creatinine levels), medical order information (drug name, medication time, and dose), and medication combinations.

Various tools from different models were used to calculate the importance value of each factor. For example, logistic regression (LR), random forest, and AdaBoost (adaptive boosting) used the eli5 Library in SK-learn to visually display the value of each feature, whereas XGBoost and LGBM used their own algorithms. We removed the features with relatively low importance to reduce the feature dimension, simplify the model, and improve its generalization ability.

### Model building

Classification algorithms in supervised learning included LR, artificial neural network, Naïve Bayes, and integration algorithms. In this study, six ML models, LR, random forest, AdaBoost, gradient boost decision tree, XGBoost, and LGBM, were established to classify and predict the blood concentration of TAC. All models except for LR belonged to the Ensemble Algorithms, which integrate several weak classifiers into one strong classifier. The Ensemble Algorithms have rapid speed and strong generalization ability, and they are suitable for application in many fields, including medical diagnosis [10].

In the process of model establishment, Grid Search was used to choose the hyperparameter of the model. Grid Search uses an exhaustive method to train the learner with the hyperparameter in the user-defined range, and then find the optimal value for the hyperparameter within this range. Table 1 lists the core hyperparameters of the six models. In addition, the threshold was continuously adjusted to achieve the best performance of the model.

**Table 1:** Hyperparameters for models

Model	Core Hyperparameters
LR	Penalty; class_weight; C
RF	min_samples_split; n_estimators; max_features; min_samples_leaf; max_depth
Adaboost	n_estimators; learning_rate
GBDT	n_estimators; learning_rate; subsample; loss; max_depth; min_samples_split; min_samples_leaf; max_features
XGBoost	learning_rate; max_depth; subsample; n_estimators; scale_pos_weight; min_child_weight; gamma
LGBM	learning_rate; max_depth; subsample; n_estimators; min_child_weight; min_child_samples; num_levels; colsample_bytree; boost_type

*LR* logistic regression; *RF* random forest; *GBDT* gradient boost decision tree; *LGBM* LightGBM

### Model assessment

The evaluation criteria of binary factors generally include accuracy, precision, recall, F-1 score, and area under the curve (AUC) and come from the confusion matrix (Table 2). Accuracy refers to the prediction accuracy of positive sample results and was calculated as follows:

1 $$\mathrm{Accuracy}=\frac{TP + TN}{TP + TN + FP + FN}$$

where TP is true positive, TN is true negative, FP is false positive, and FN is false negative.

**Table 2:** Confusion matrix

	Predicted: True	Predicted: False
Actual: True	True Positive (TP)	False Negative (FN)
Actual: False	False Positive (FP)	True Negative (TN)

*FN* positive class is judged as negative (type I error); *FP* negative class is judged as positive (type II error)

Recall refers to how many positive samples in the data set are identified and can be calculated as follows:

2 $$\mathrm{Recall} =\frac{TP}{TP + FN}$$

In the ideal state, accuracy and recall are as high as possible; however, the two factors are inversely related, and a balance must be achieved. Therefore, the F-beta score was used to reflect the comprehensive situation of the model. The F-beta score was calculated using the following formula:

3 $$F - beta = 1 + \beta^{2} \times \frac{Precision \times Recall }{{\beta^{2} \times Precision + Recall}}$$

where precision is calculated using Eq. 4, $\beta$ equals 1, and the F-beta score is calculated using Eq. 5.

4 $$\mathrm{Precision} =\frac{TP}{TP + FP}$$

5 $$\mathrm{F}1=2\times \frac{Precision \times Recall}{Precision +Recall}$$

When the accuracy and recall are equally important, they are given the same weight, that is, beta = 1 (F-1 score). However, in this study, type II errors were particularly important. Thus, we closely monitored situations in which patients with abnormal blood concentrations were not assessed, which had a negative effect on the treatment outcomes. Type II errors were generally measured by recall. Therefore, in this study, greater weight was given to recall, where beta = 2 (F-2 score). The F-beta score was > 0 and < 1, and the larger the value, the better the performance of the model. Finally, when the AUC was > 0.5, the model was meaningful. AUC can be calculated as follows:

6 $$\mathrm{AUC} =\frac{1 + TPR - FPR}{2}$$

where true positive rate (TPR) and false positive rate (FPR) are calculated using Eqs. 7 and 8, respectively.

7 $$\mathrm{TPR} =\frac{TP}{TP + FN}$$

8 $$\mathrm{FPR} =\frac{FP}{FP + TN}$$

## Results

### Baseline information

Data from 913 patients and 1829 blood tests were included in this study. The baseline information of the study population is shown in Table 3. Continuous variables are presented as median (interquartile range [IQR]) and categorical variables as frequency (percentage). The median age of the patients in this study was 53 (39–64) years, median weight was 72 (64–80) kg, median height was 170 (162–174) cm, median serum creatinine level was 80.9 (65.8–103.1) μmol/L, and proportion of male patients was 66%. Additionally, the proportion of combined medication was as follows: 8.64% for compound α-ketoacid, 6.01% for Shenyankangfu, 12.30% for ShenYanShu, 6.51% for Shenshuaining, 45.05% for Huangkui, 39.58% for Bailing, 46.53% for pidotimod, 29.36% for methylprednisolone, 16.07% for prednisone acetate, 1.04% for mycophenolate mofetil, and 2.35% for tripterygium glycoside.

**Table 3:** Baseline information

Feature	*n*	(%)
*Independent Variable*		
Blood Concentration of TAC, n, Class 1	1292	70.64
Class 0	537	29.36
*Demographic Information*		
Age, yr, median (IQR)	53 (39, 64)	–
Weight, kg, median (IQR)	72 (64, 80)	–
Height, cm, median (IQR)	170 (162, 174)	–
FK506, mg, median (IQR)	1.5 (1, 2)	–
Sex, Male	1241	67.85
Female	588	32.15
*Laboratory Information*		
SC, μmol/L, median (IQR)	80.9 (65.8, 103.1)	–
*Combined Medication*		
CαK, n, Class 1	158	8.64
Class 0	1671	91.36
SYKF, n, Class 1	110	6.01
Class 0	1719	93.99
SYS, n, Class 1	225	12.30
Class 0	1604	87.70
SSN, n, Class 1	119	6.51
Class 0	1710	93.49
HK, n, Class 1	824	45.05
Class 0	1005	54.95
BL, n, Class 1	724	39.58
Class 0	1105	60.42
Pidotimod, n, Class 1	851	46.53
Class 0	978	53.47
MPS, n, Class 1	537	29.36
Class 0	1292	70.64
PA, n, Class 1	294	16.07
Class 0	1535	83.93
MM, n, Class 1	19	1.04
Class 0	1810	98.96
TG, n, Class 1	43	2.35
Class 0	1786	97.65

*SC* serum creatinine; *CαK* compound α-ketoacid; *SYKF* Shenyankangfu; *SYS* ShenYanShu; *SSN* Shenshuaining; *HK* Huangkui; *BL* Bailing; *MPS* methylprednisolone; *PA* prednisone acetate; *MM* mycophenolate mofetil; *TG* tripterygium glycoside

### Model performance

The prediction performance of the six models is shown in Table 4. In terms of accuracy, only XGBoost and LGBM displayed an accuracy of > 70%; the accuracy of XGBoost was higher than that of LGBM at 73.33%. The accuracy of the other models was low, and the effect was poor. We evaluated type II errors through the recall rate. A higher recall rate means that more patients with abnormal blood drug concentrations were correctly predicted, and clinicians can therefore adjust the dosage to reach effective and safe blood drug concentrations. However, when the probability of type II errors was low, the probability of type I errors increased. Therefore, XGBoost performed the best in balancing type I and II errors (F-beta score = 0.9124). In addition, the AUC value of XGBoost was the highest among all models. Therefore, considering the generalization ability and accuracy of the model, we believe that the XGBoost model is ideal for predicting the blood concentration of TAC.

**Table 4:** Performance of the models

Metrics model	RF	LR	AdaBoost	GBDT	XGBoost	LGBM
Accuracy	0.6556	0.5444	0.6667	0.6556	0.7333	0.7167
F-beta	0.8221	0.5810	0.8638	0.7967	0.9124	0.9075
Recall	0.8527	0.5504	0.9147	0.8140	0.9690	0.9165
Precision	0.7190	0.7474	0.7066	0.7343	0.7396	0.8730
AUC	0.5048	0.5399	0.4770	0.5344	0.5531	0.4947

*LR* logistic regression; *RF* random forest; *GBDT* gradient boost decision tree; *LGBM* LightGBM; *AUC* area under the curve

### Feature analysis

Table 5 shows the performance of the XGBoost model under different quantitative features. The features were selected from top to bottom according to the feature importance of the XGBoost model. Although the recall rate of the model was 1 when the number of features in the model was three or less, the AUC was only 0.5, and the model was extremely poor with no effective discriminative ability. Thus, very few features will lead to the underfitting of the model. With an increase in the number of features during modeling, the evaluation indexes in Table 5 increased even if they slightly fluctuated. When the number of features was eight, all evaluation indexes were maximized (accuracy = 0.7333, F-beta = 0.9124, and AUC = 0.5531), and the performance of the model was the best. When the number of features increased beyond eight, the evaluation indexes decreased overall. Thus, too many features weakened the generalization ability of the model, causing the overfitting phenomenon. Therefore, the performance of the model was optimized when using the top eight features for modeling.

**Table 5:** Performance comparison of models according to number of features

No. of Features	1	2	3	4	5	6	7	8	9	10	11	12	13	14	15	16	17
Accuracy	0.7167	0.7167	0.7167	0.7056	0.7000	0.7056	0.7111	0.7333	0.7258	0.7268	0.7278	0.7167	0.7222	0.7167	0.7278	0.7333	0.7222
F-beta	0.9267	0.9267	0.9267	0.9011	0.9045	0.8823	0.9071	0.9124	0.9102	0.9093	0.9064	0.9038	0.9051	0.8944	0.9064	0.9170	0.9143
Recall	1.0000	1.0000	1.0000	0.9612	0.9690	0.9302	0.9690	0.9690	0.9664	0.9745	0.9612	0.9612	0.9612	0.9457	0.9612	0.9767	0.9767
Precision	0.7167	0.7167	0.7167	0.7209	0.7143	0.7317	0.7225	0.7396	0.7384	0.7174	0.7381	0.7294	0.7337	0.7349	0.7381	0.7368	0.7283
AUC	0.5000	0.5000	0.500	0.5100	0.4943	0.5337	0.5139	0.5531	0.5374	0.5315	0.5492	0.5296	0.5394	0.5415	0.5492	0.5472	0.5276

*AUC* area under the curve

**Fig. 4:**
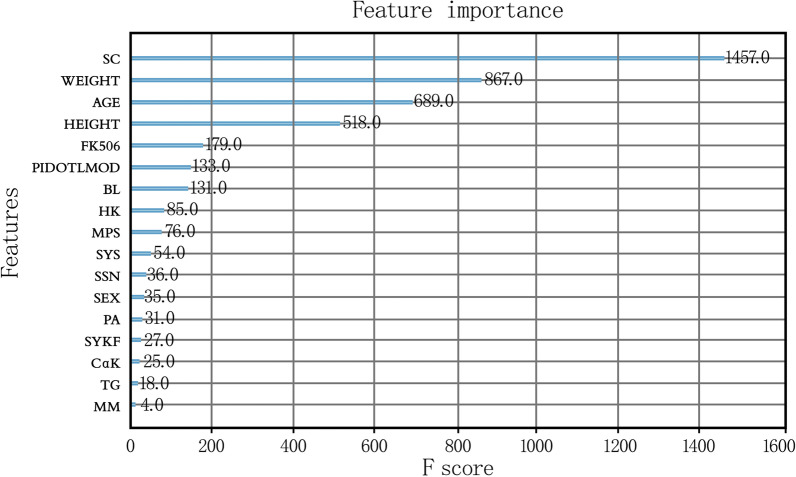
Feature importance of the XGBoost model

As shown in Fig. 4, the top eight features in the XGBoost model in descending order were serum creatinine level, weight, age, height, TAC dosage, pidotimod, Bailing, and Huangkui usage. Among them, serum creatinine level was nearly twice as important as any other feature, indicating that serum creatinine has a significant effect on the blood concentration of TAC. Weight, age, and height were also more important than many other characteristics, whereas sex and some combined medications had relatively little influence on the model.

## Discussion

This study revealed that the XGBoost model—with an accuracy of 0.7333 and an F-beta score of 0.9124—showed the best effect that could be used to monitor the blood concentration of TAC. Zheng et al. [11] also achieved the best results in regression prediction of TAC blood concentration from real-world data using the XGBoost model. Thus, the XGBoost model has certain advantages for clinical data prediction in real-world settings.

The feature importance ranking of XGBoost revealed that the serum creatinine level of the patients with kidney diseases, particularly nephrotic syndrome and membranous nephropathy, had a significant effect on their blood TAC concentration, thus confirming that the blood TAC concentration is positively correlated with the serum creatinine level [12]. Weight and height also ranked high, in this study, as factors that affect the blood TAC concentration, which is consistent with the results from Zheng et al. [11, 13]. Patient age is routinely evaluated by researchers [14, 15]. In this study, it ranked third among all features. Finally, the importance of sex in the prediction model was relatively low and did not participate in the establishment of the final model.

Previous studies have focused on the effect of TAC combined with other drugs [16, 17], but did not evaluate the effect of the combination on blood TAC concentration. Our study showed that the combination of Bailing and Huangkui with TAC affects blood TAC concentration. However, although pidotimod also had a high importance value in our study, there are no reports to support this result. It is speculated that it may be related to the medication habits of physicians. These conclusions warrant future research.

In the last decade, a few studies have described the prediction of TAC concentration in the blood using ML technology. Additionally, the models used in previous research were mostly artificial neural networks and regression models [18, 19], the amount of data obtained was lower, the models were not verified externally, and the research is still in the exploratory stage. In this study, using patients with nephrotic syndrome and membranous nephropathy as examples, blood TAC concentration was classified according to the safe blood concentration range and predicted using a variety of ML models. The number of real-world samples included in this study was considerably more than that in previous research, and an external validation set was used to verify the model. Thus, the model results are more authentic and have clinical significance over previous models.

This study had several limitations. First, owing to the lack of information about blood sample collection time, we had to use the sample-receiving time. Ideally, the laboratory department can obtain the sample collection time in the future to further strengthen the integrity and analyzability of medical data. Second, more laboratory and genetic data should be analyzed.

## Conclusion

In this study, an ML model was established to classify the blood TAC concentration in patients with nephrotic syndrome and membranous nephropathy. The over-sampling method was used to manage unbalanced data, the variables were screened according to their importance value, and the performance of the six models was compared. Finally, XGBoost was selected as the best prediction model, considering its accuracy of 0.7333, F-beta score of 0.9124, and AUC of 0.5531, which were higher than those of other models, demonstrating a better prediction ability. In the XGBoost model, serum creatinine, weight, age, height, TAC dose, and the use of pidotimod, Bailing, and Huangkui were the main influencing factors of blood TAC concentration. The low AUC and high sensitivity of the model also implies that it is biased towards positive class, which may have a negative impact on the prediction of clinical dose of TAC in patients with negative class. In this exploratory study, the ability of machine learning in predicting TAC blood concentration was investigated. The study findings prove the predictive potential of machine learning to a certain extent; however, further in-depth research is needed to resolve the model’s bias towards positive class.

## Data Availability

The data that support the findings of this study are available from the corresponding author upon reasonable request.

## Abbreviations

SC: Serum creatinine
CαK: Compound α-ketoacid
SYKF: Shenyankangfu
SYS: ShenYanShu
SSN: Shenshuaining
HK: Huangkui
BL: Bailing
MPS: Methylprednisolone
PA: Prednisone acetate
MM: Mycophenolate mofetil
TG: Tripterygium glycoside
ML: Machine learning
LR: Logistic regression
AUC: Area under the curve
RF: Random forest
GBDT: Gradient boost decision tree
LGBM: LightGBM

## References

[1] Kohli HS, Rajachandran R, Rathi M, Jha V, Sakhuja V (2013). Tacrolimus in nephrotic syndrome resistant to first line therapy in adults: a prospective study. Nephrol Dial Transplant.

[2] Zhang J, Zhang Y, Yang H (2015). Effect of tacrolimus on renal function, blood lipids, cytokines and peripheral HMGB-1 and NF-κB in nephrotic syndrome patients. Chin J Biochem Pharm.

[3] Gérard C, Stocco J, Hulin A, Blanchet B, Verstuyft C, Durand F (2014). Determination of the most influential sources of variability in tacrolimus trough blood concentrations in adult liver transplant recipients: a bottom-up approach. AAPS J.

[4] Koelzer VH, Sirinukunwattana K, Rittscher J, Mertz KD (2019). Precision immunoprofiling by image analysis and artificial intelligence. Virchows Arch.

[5] Schork NJ (2019). Artificial intelligence and personalized medicine. Cancer Treat Res.

[6] Shamout F, Zhu T, Clifton DA (2021). Machine learning for clinical outcome prediction. IEEE Rev Biomed Eng.

[7] Santosh T, Liu H, Liu B (2014). Effect of tacrolimus in idiopathic membranous nephropathy: a meta-analysis. Chin Med J.

[8] Liang Q, Li H, Xie X, Qu F, Li X, Chen J (2017). The efficacy and safety of tacrolimus monotherapy in adult-onset nephrotic syndrome caused by idiopathic membranous nephropathy. Ren Fail.

[9] Dong H, He D, Wang F (2020). SMOTE-XGBoost using Tree Parzen Estimator optimization for copper flotation method classification. Powder Technol.

[10] Dong X, Yu Z, Cao W, Shi Y, Ma Q (2020). A survey on ensemble learning. Front Comput Sci.

[11] Zheng P, Yu Z, Li L, Liu S, Lou Y, Hao X (2021). Predicting blood concentration of tacrolimus in patients with autoimmune diseases using machine learning techniques based on real-world evidence. Front Pharmacol.

[12] Jing-ge G. 郭景鸽. Chin Rem Clin. Takemosi zhiliao chengren jisu dikangxing shenbing zonghezheng xueyao nongdu yu linchuang xiaoguo ji buliang fanying de xiangguanxing yanjiu 他克莫司治疗成人激素抵抗型肾病综合征血药浓度与临床效果及不良反应的相关性分析 [Correlation analysis between blood concentration, clinical effect, and adverse reactions of tacrolimus in the treatment of the adult hormone-resistant nephrotic syndrome]. 2019;19:773–5.

[13] Sam WJ, Tham LS, Holmes MJ, Aw M, Quak SH, Lee KH (2006). Population pharmacokinetics of tacrolimus in whole blood and plasma in Asian liver transplant patients. Clin Pharmacokinet.

[14] Przepiorka D, Blamble D, Hilsenbeck S, Danielson M, Krance R, Chan KW (2000). Tacrolimus clearance is age-dependent within the pediatric population. Bone Marrow Transplant.

[15] Staatz CE, Tett SE (2005). Pharmacokinetic considerations relating to tacrolimus dosing in the elderly. Drugs Aging.

[16] Yan Xiao-hui LY, Feng Ting JG, Xiao-Ming W (2017). Clinical effects of tacrolimus combined with okra capsule in treatment of refractory membranous nephropathy. Prog Mod Biomed.

[17] Li Y, Xu T, Qiu X, Tian B, Bi C, Yao L (2020). Effectiveness of Bailing capsules in the treatment of lupus nephritis: a meta-analysis. Mol Med Rep.

[18] Venkataramanan R, Shaw LM, Sarkozi L, Mullins R, Pirsch J, MacFarlane G (2001). Clinical utility of monitoring tacrolimus blood concentrations in liver transplant patients. J Clin Pharmacol.

[19] Tang J, Liu R, Zhang YL, Liu MZ, Hu YF, Shao MJ (2017). Application of machine-learning models to predict tacrolimus stable dose in renal transplant recipients. Sci Rep.

